# Supplementary material for: GENESIS CGDYN: large-scale coarse-grained MD simulation with dynamic load balancing for heterogeneous biomolecular systems
Source: Nat Commun. 2024 Apr 20;15:3370. doi: 10.1038/s41467-024-47654-1 (PMC11032353; doi:10.1038/s41467-024-47654-1)
Supplement: Supplementary file 1 — Supplementary Information [file 41467_2024_47654_MOESM1_ESM.pdf]

SUPPLEMENTARY INFORMATION

GENESIS CGDYN: large-scale coarse-grained MD simulation with dynamic load balancing for heterogeneous biomolecular systems

Jaewoon Jung,[1,2,*] Cheng Tan,[1*] Yuji Sugita[1,2,3,#]

1. Computational Biophysics Research Team, RIKEN Center for Computational Science, Kobe, Hyogo 650-0047, Japan

2. Theoretical Molecular Science Laboratory, RIKEN Cluster for Pioneering Research, Wako, Saitama 351-0198, Japan

3. Laboratory for Biomolecular Function Simulation, RIKEN Center for Biosystems Dynamics Research, Kobe, Hyogo 650-0047, Japan

*: equally contributed authors

#: corresponding author, email: sugita@riken.jp

Supplementary Figures

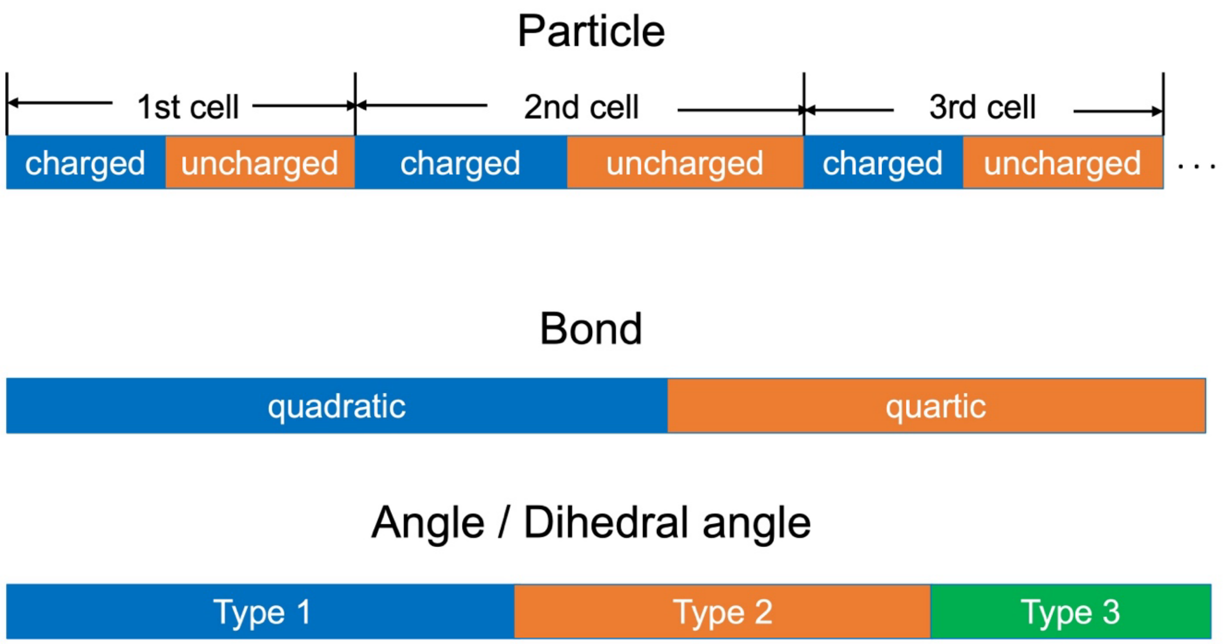

Supplementary Figure 1. Array structures of the coordinates, bonds, and angles/dihedral angles. In each cell, we save all the charged and uncharged particles in this order. Bond/Angle/Dihedral angle and other bonded interaction data are not saved cell-wise. There are multiple potential functions for the same bonded term. We save the same potential function terms consecutively.

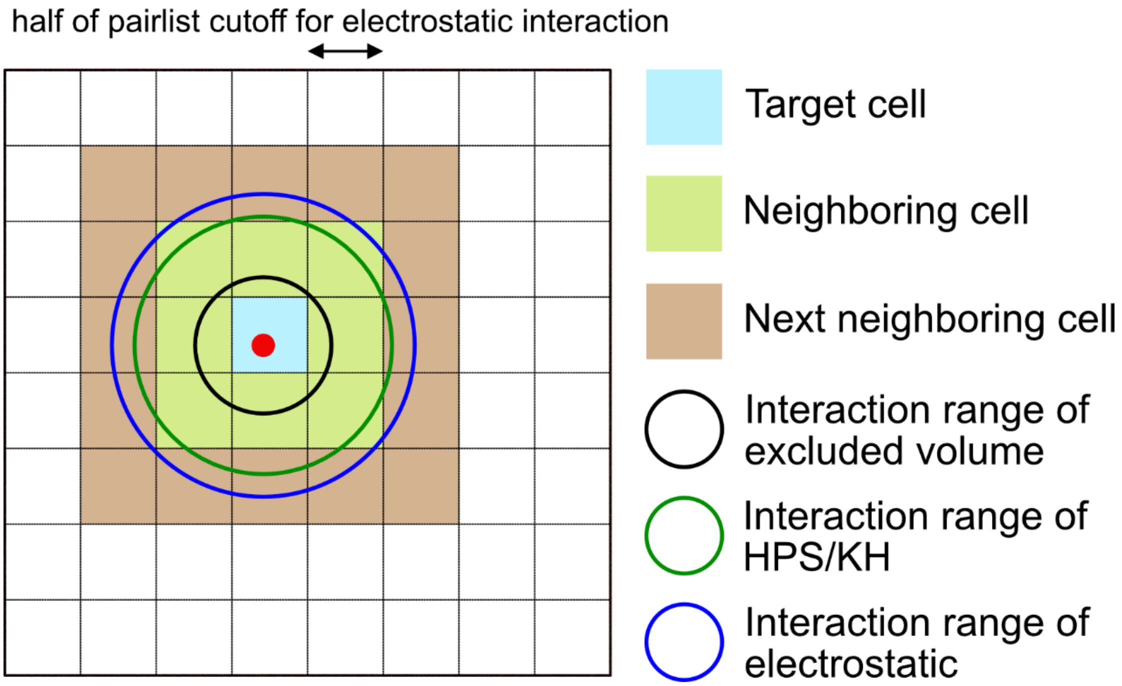

Supplementary Figure 2. Interaction ranges of potential functions. For a particle marked as a red circle, the interaction ranges of the excluded volume, HPS, and electrostatic potentials are represented as black, green and blue circles, respectively. Because of the short interaction range of the excluded volume, we can consider only particles in the cells, including the particles in the target cell and those in the neighboring cells. The interaction range of HPS/KH and electrostatic is much longer, and we consider the particles in the next neighboring cells as well as the target/neighboring cells.

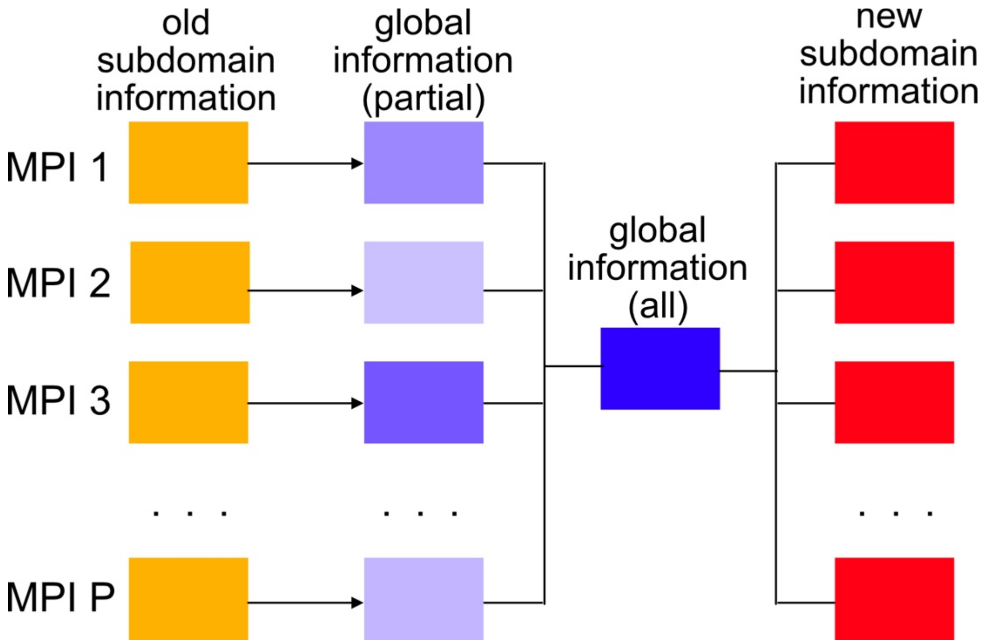

Supplementary Figure 3. Cell reassignment in dynamic load balancing. First, each process saves its subdomain data into a global information array. After collective communications, all processes share the global information data of all particles and redefine subdomains based on the particle densities from the global information data.

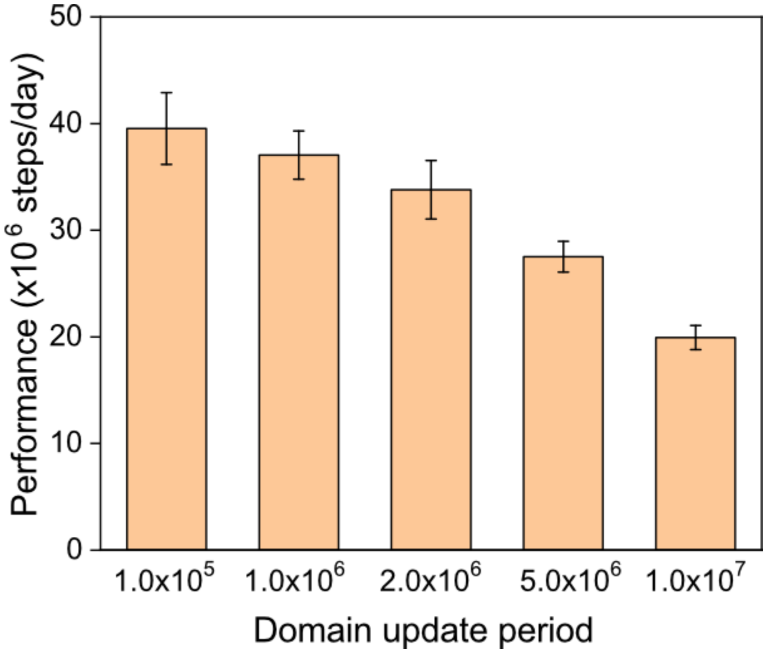

Supplementary Figure 4. Performance of droplet systems by changing the domain update period. Data are presented as mean values +/- standard deviation. Error bars indicate the standard deviation, based on n=5 independent simulations. Source data are provided as a Source Data file.

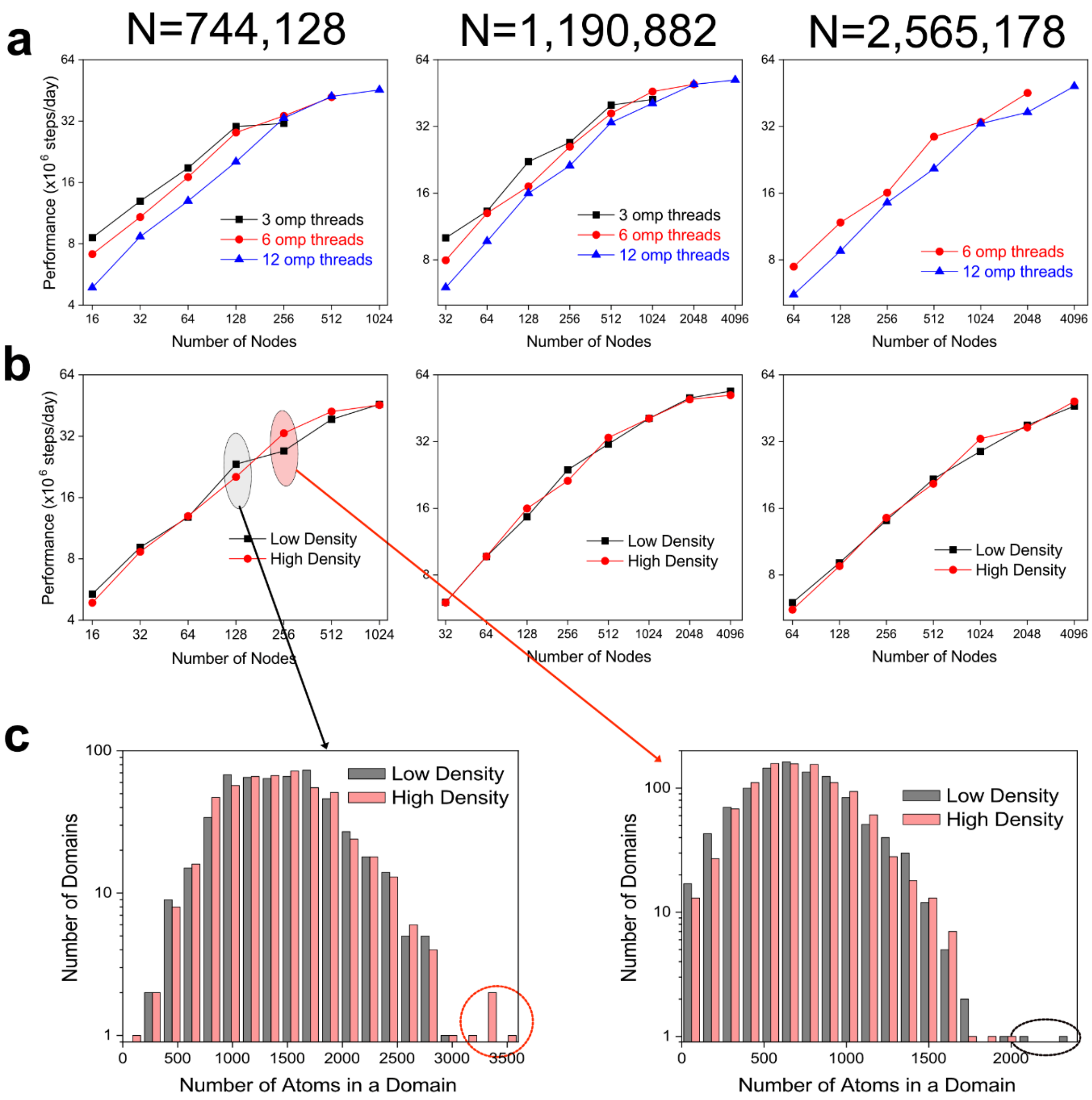

Supplementary Figure 5. Benchmark results of droplet systems in CG MD simulations with CGDYN. (a) Benchmark performance of a high-density system as a function of the number of nodes, changing the number of OpenMP threads. (b) Comparison of the performances between the high- and low-density systems. There is no significant performance difference between them. (c) The distributions of the number of particles in domains with CGDYN. The performance is limited by the maximum number of particles in a domain. Source data are provided as a Source Data file.

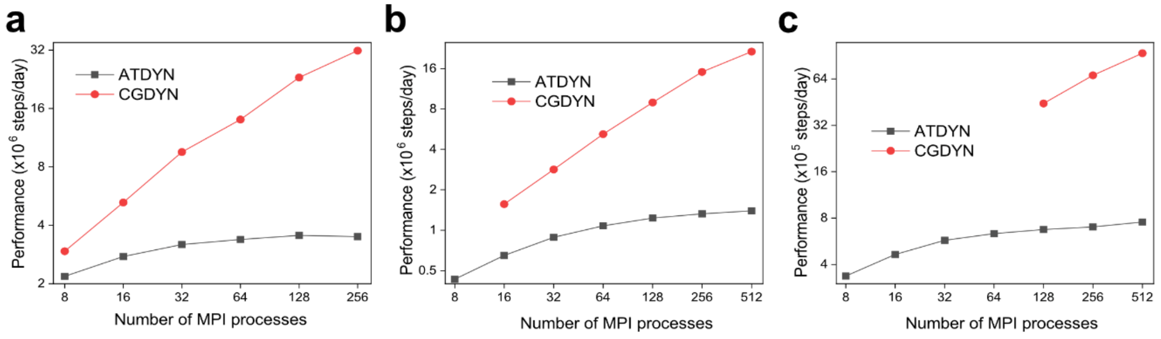

Supplementary Figure 6. Performance of MD simulations using CGDYN for (a) 120 DPS proteins (222,360 particles), (b) 5,000 chains of 100 amino acid IDPs (500,000 particles), and (c) 512 nucleosomes (1,044,480 particles) on the RIKEN Hokusai supercomputer. Source data are provided as a Source Data file.

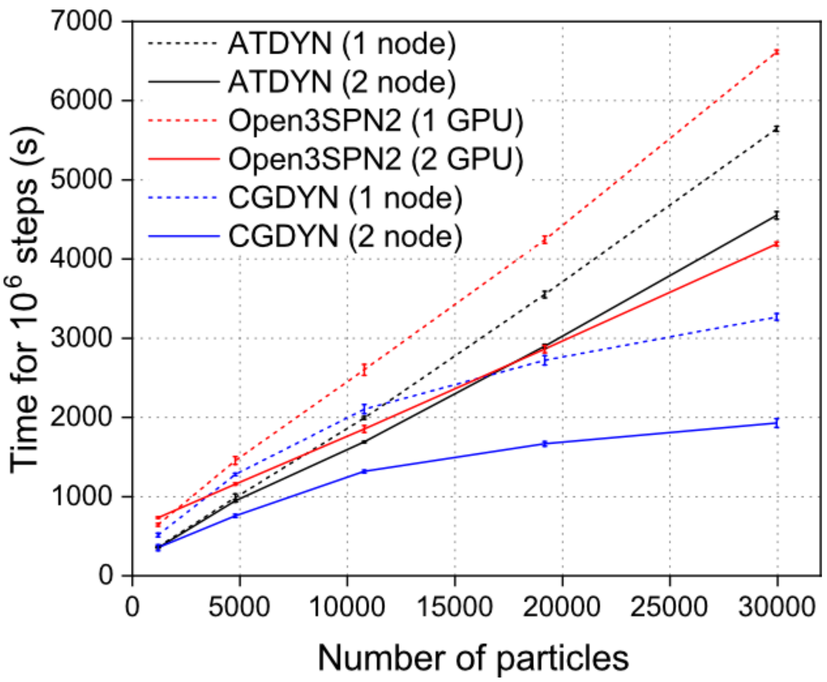

Supplementary Figure 7. Comparison of the performance of multiple DNA chains. For a very small system, ATDYN shows the best performance. As the system size increases, CGDYN shows better performance than ATDYN and Open3SPN2. CGDYN has the best performance irrespective of the system size, just using 2 nodes. Data are presented as mean values +/- standard deviation. Error bars indicate the standard deviation, based on n=5 independent simulations. Source data are provided as a Source Data file.

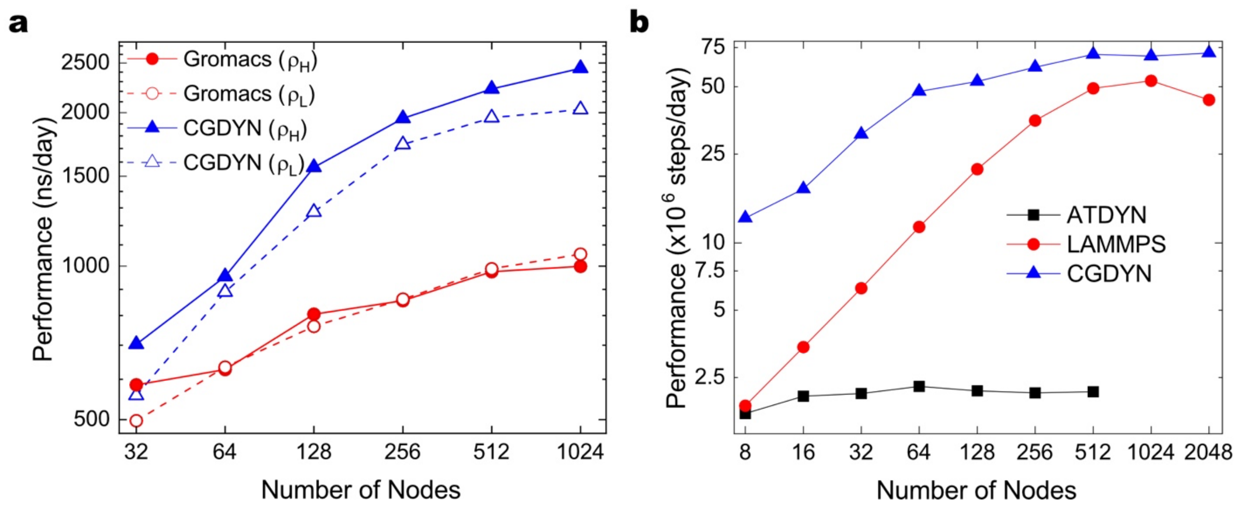

Supplementary Figure 8. Benchmark performance of (a) CGDYN and GROMACS for 1000 DPPC micelle clusters (1.2 million particles) with high and low densities, and (b) ATDYN, CGDYN, and LAMMPS for droplet system using HPS potential. Source data are provided as a Source Data file.

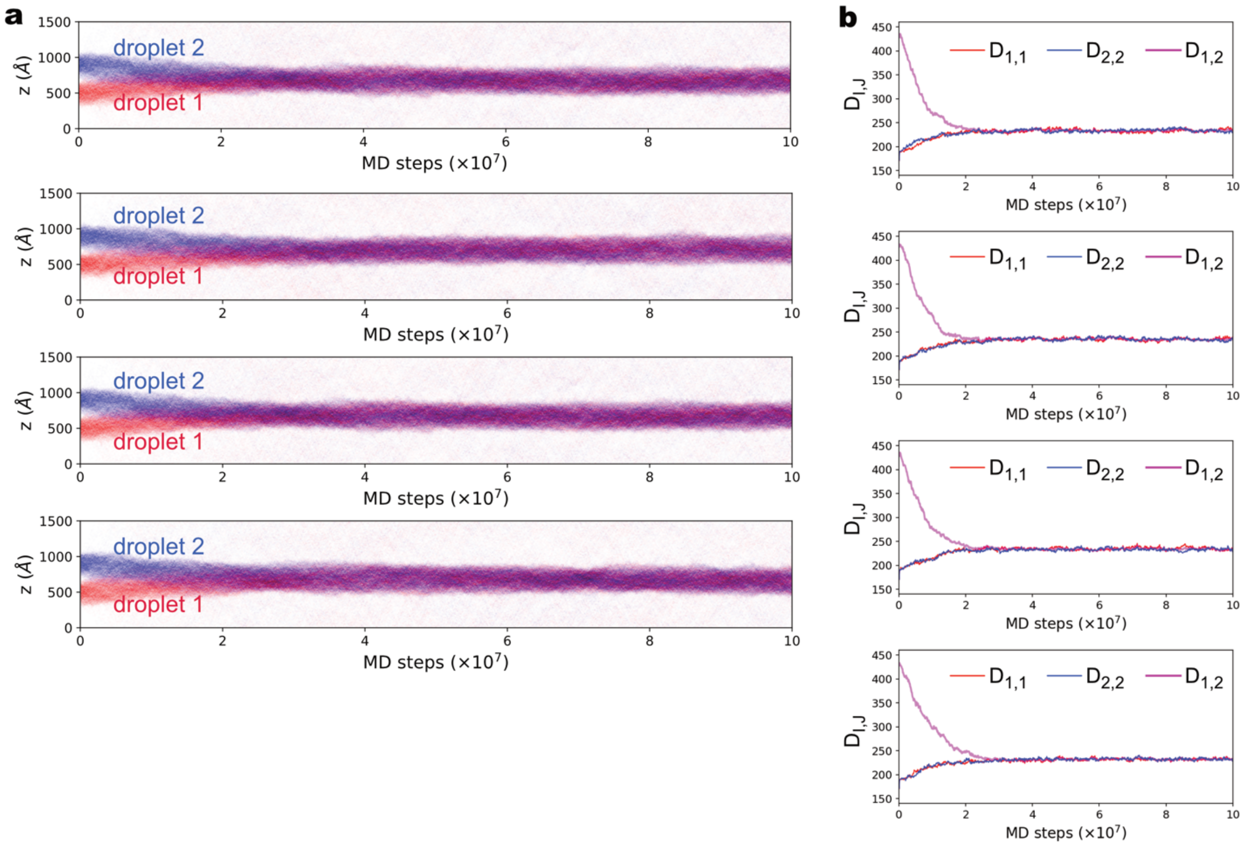

Supplementary Figure 9. The other four CG MD simulations of the fusion process of two TDP-43-LCD droplets. These results are analyzed from simulations same as Fig.3 but with different initial structures and random number seeds. (a) Density of TDP-43 particles along the z axis as a function of simulation time. (b) Time series of average chain-chain distances $D_{I,J}$. Source data are provided as a Source Data file.

Supplementary Tables

Supplementary Table 1. Benchmark performance of multiple DNAs (unit is 10^6 steps/day).

Number of Nodes	Number of particles = 119,800		Number of particles = 269,550		Number of particles = 479,200	
	ATDYN	CGDYN	ATDYN	CGDYN	ATDYN	CGDYN
1	3.576		1.475		0.807	
2	4.557		1.916		1.056	
4	5.437		2.294		1.247	
8	3.576	15.393	2.509	10.363	1.380	
16	6.278	20.433	2.728	17.728	1.520	9.876
32	5.922	29.993	2.739	27.078	1.560	13.789
64	5.548	42.667	2.743	42.562	1.617	20.241
128	4.192	44.158	2.494	40.393	1.506	25.681

Supplementary Methods

Supplementary Algorithm. Neighbor List generation in CGDYN

---

for $C_\alpha \in D_k \cup B_k$ **do**

  for $i_\alpha \in C_\alpha$ **do**

    $\vec{r}_{i_\alpha}, t_{i_\alpha}$ ▷ coordinate, particle type

    for $C_\beta \in$ neighbor(C_α) **do**

      for $j_\beta \in C_\beta$ **do**

        $\vec{r}_{j_\beta},, t_{j_\beta}$

        $r_{i_\alpha j_\beta}$ ▷ pairwise distance

        if $(r_{i_\alpha j_\beta} < r_{\mathrm{p,exv}})$ **do**

          write neighobr list of exclude volume, DNA base pairing and HPS/KH

        else if $(r_{i_\alpha j_\beta} < r_{\mathrm{p,dna}})$ **do**

          write neighobr list of DNA base pairing and HPS/KH repulsion

        else if $(r_{i_\alpha j_\beta} < r_{\mathrm{p,hps}})$ **do**

          write neighobr list of HPS/KH repulsion

D_k: Subdomain region of the k-th subdomain

B_k: Adjacent cell region of the k-th subdomain

C_α: Cell indices of the α-th cell

i_α: Particle indices of i-th atom in the α-th cell

r_{i_α}: Coordinate of i-th atom in the α-th cell

t_{i_α}: Particle type of i-th atom in the α-th cell
