# Supplementary material for: GENESIS CGDYN: large-scale coarse-grained MD simulation with dynamic load balancing for heterogeneous biomolecular systems
Source: Nat Commun. 2024 Apr 20;15:3370. doi: 10.1038/s41467-024-47654-1 (PMC11032353; doi:10.1038/s41467-024-47654-1)
Supplement: Supplementary file 3 — Description of Additional Supplementary Files [file 41467_2024_47654_MOESM3_ESM.pdf]

Description of Additional Supplementary Files

File Name: Supplementary Movie 1
Description: MD simulation of the fusion process of two droplets. We used HPS (for intrinsically disordered regions) and AICG2+ (for α-helix) models. The system consists of 1000 TDP-43-LCD chains and is simulated at 280K. The movie corresponds to 10^8 steps. The simulation box size is 1000Å×1000Å×1500Å with periodic boundary conditions. Chains from the two initial droplets are colored red and blue, respectively. See Figure 3 for more quantitative analysis results.

File Name: Supplementary Movie 2
Description: MD simulations of multiple TDP-43-LCD droplets. The system comprises 16,657 proteins and is simulated at 290K using the HPS (for intrinsically disordered regions) and AICG2+ (for α-helix) models. The simulation box size is 2087Å×2067Å×2077Å with periodic boundary conditions. The movie corresponds to 12.25×10^8 MD steps. Chains are colored based on clustering results for each frame. See Figure 4 for more quantitative analysis results.
