# Supplementary material for: GENESIS CGDYN: large-scale coarse-grained MD simulation with dynamic load balancing for heterogeneous biomolecular systems
Source: Nat Commun. 2024 Apr 20;15:3370. doi: 10.1038/s41467-024-47654-1 (PMC11032353; doi:10.1038/s41467-024-47654-1)
Supplement: Supplementary file 6 — Reporting Summary [file 41467_2024_47654_MOESM6_ESM.pdf]

nature portfolio

Corresponding author(s): Yuji Sugita

Last updated by author(s): Apr 1, 2024

Reporting Summary

Nature Portfolio wishes to improve the reproducibility of the work that we publish. This form provides structure for consistency and transparency in reporting. For further information on Nature Portfolio policies, see our Editorial Policies and the Editorial Policy Checklist.

Statistics

For all statistical analyses, confirm that the following items are present in the figure legend, table legend, main text, or Methods section.

n/a	Confirmed	
☐	☒	The exact sample size (*n*) for each experimental group/condition, given as a discrete number and unit of measurement
☐	☒	A statement on whether measurements were taken from distinct samples or whether the same sample was measured repeatedly
☒	☐	The statistical test(s) used AND whether they are one- or two-sided<br>*Only common tests should be described solely by name; describe more complex techniques in the Methods section.*
☒	☐	A description of all covariates tested
☐	☒	A description of any assumptions or corrections, such as tests of normality and adjustment for multiple comparisons
☐	☒	A full description of the statistical parameters including central tendency (e.g. means) or other basic estimates (e.g. regression coefficient) AND variation (e.g. standard deviation) or associated estimates of uncertainty (e.g. confidence intervals)
☒	☐	For null hypothesis testing, the test statistic (e.g. *F*, *t*, *r*) with confidence intervals, effect sizes, degrees of freedom and *P* value noted<br>*Give P values as exact values whenever suitable.*
☒	☐	For Bayesian analysis, information on the choice of priors and Markov chain Monte Carlo settings
☒	☐	For hierarchical and complex designs, identification of the appropriate level for tests and full reporting of outcomes
☒	☐	Estimates of effect sizes (e.g. Cohen's *d*, Pearson's *r*), indicating how they were calculated

Our web collection on statistics for biologists contains articles on many of the points above.

Software and code

Policy information about availability of computer code

Data collection	1. The MD simulations were performed using the developer version of GENESIS MD software, version 2.1, which is available for download at https://github.com/genesis-release-r-ccs/genesis-2.1.0beta_cgdyn<br>2. Figures were created using vmd1.9.4a57 (for protein structures), origin2023 (for graphs), matplotlib3.7 (for graphs) and Affinity Designer 2.1.1 (for vector graphics editing).
Data analysis	We used the following programs for data analysis:<br>1. Developer version of GENESIS MD software 2.1, available at: https://github.com/genesis-release-r-ccs/genesis-2.1.0beta_cgdyn<br>2. GENESIS-CG-tool, accessible at: https://github.com/genesis-release-r-ccs/genesis_cg_tool<br>3. In-house scripts, found at: https://github.com/RikenSugitaLab/cgdyntest/

For manuscripts utilizing custom algorithms or software that are central to the research but not yet described in published literature, software must be made available to editors and reviewers. We strongly encourage code deposition in a community repository (e.g. GitHub). See the Nature Portfolio guidelines for submitting code & software for further information.

Data

Policy information about availability of data

All manuscripts must include a data availability statement. This statement should provide the following information, where applicable:

- Accession codes, unique identifiers, or web links for publicly available datasets
- A description of any restrictions on data availability
- For clinical datasets or third party data, please ensure that the statement adheres to our policy

All input datasets for benchmarks and MD simulations, as well as data analysis programs and scripts, are available at https://github.com/RikenSugitaLab/cgdyntest/. Regarding the MD trajectory, due to our system's extra-large scales, the total output trajectory amounts to 7.0 TB. Therefore, we have provided the initial and final structures, along with several intermediate structures from the simulations (found in the same git repository). Additionally, our trajectories are available upon request.

Research involving human participants, their data, or biological material

Policy information about studies with human participants or human data. See also policy information about sex, gender (identity/presentation), and sexual orientation and race, ethnicity and racism.

Reporting on sex and gender	N/A
Reporting on race, ethnicity, or other socially relevant groupings	N/A
Population characteristics	N/A
Recruitment	N/A
Ethics oversight	N/A

Note that full information on the approval of the study protocol must also be provided in the manuscript.

Field-specific reporting

Please select the one below that is the best fit for your research. If you are not sure, read the appropriate sections before making your selection.

☒ Life sciences ☐ Behavioural & social sciences ☐ Ecological, evolutionary & environmental sciences

For a reference copy of the document with all sections, see nature.com/documents/nr-reporting-summary-flat.pdf

Life sciences study design

All studies must disclose on these points even when the disclosure is negative.

Sample size	1. We repeated each benchmark simulation five times and confirmed that the standard deviation across these calculations is minimal, indicating reliable consistency.<br>2. In the application MD simulations for the two-droplet systems, we conducted five independent runs with different random seeds, each comprising 10^8 MD steps. These simulations were extensive enough to ensure equilibrium, as evidenced by Fig. 3g. For the more complex multiple droplet systems, we extended our simulations to exceed 1.2x10^9 MD steps. This extraordinarily long MD simulations enabled us to observe large-scale phase behaviors in biomolecular droplets, previously unachieved in MD simulations.
Data exclusions	No data has been excluded.
Replication	This study focuses on MD software development and computer simulations, thus replication was ensured within the simulation framework.<br>1. For benchmarks, each was repeated five times, confirming that the standard deviations of the results were sufficiently small.<br>2. For application simulations of small droplet systems, we initiated multiple independent simulations from varied initial structures, yielding consistent outcomes. Additional simulations were conducted to further guarantee reproducibility whenever applicable.
Randomization	This study encompasses MD software development and computer simulations.<br>The development process itself does not involve randomness. However, in both the benchmark and application simulations, we introduced random forces through the use of Langevin dynamics. Additionally, for the MD simulations, we prepared the initial structures by generating independent random configurations of protein droplets.
Blinding	Blinding was not performed as it is not relevant to this computer simulation study.

Reporting for specific materials, systems and methods

We require information from authors about some types of materials, experimental systems and methods used in many studies. Here, indicate whether each material, system or method listed is relevant to your study. If you are not sure if a list item applies to your research, read the appropriate section before selecting a response.

Materials & experimental systems

n/a	Involved in the study
☒	☐ Antibodies
☒	☐ Eukaryotic cell lines
☒	☐ Palaeontology and archaeology
☒	☐ Animals and other organisms
☒	☐ Clinical data
☒	☐ Dual use research of concern
☒	☐ Plants

Methods

n/a	Involved in the study
☒	☐ ChIP-seq
☒	☐ Flow cytometry
☒	☐ MRI-based neuroimaging
